# Invasive pulmonary aspergillosis in cirrhotic patients: analysis of a 10-year clinical experience

**DOI:** 10.1186/s13613-019-0502-2

**Published:** 2019-02-18

**Authors:** Eric Levesque, Nawel Ait-Ammar, Daniela Dudau, Noémie Clavieras, Cyrille Feray, Françoise Foulet, Françoise Botterel

**Affiliations:** 1 Department of Anaesthesia and Surgical Intensive Care-Liver ICU, AP-HP Henri Mondor Hospital, 51 avenue du Maréchal de Lattre de Tassigny, 94010 Créteil, France; 2 Ecole Nationale Vétérinaire d’Alfort (ENVA), Faculté de Médecine de Créteil, EA Dynamyc Université Paris-Est Créteil (UPEC), 8 rue du Général Sarrail, 94010 Créteil, France; 3 Mycology Unit-Microbiology Department DHU, AP-HP Henri Mondor Hospital, 51 avenue du Maréchal de Lattre de Tassigny, 94010 Créteil, France; 4 Hepatology Department, AP-HP Henri Mondor Hospital, 51 avenue du Maréchal de Lattre de Tassigny, 94010 Créteil, France

**Keywords:** Aspergillus, Invasive pulmonary aspergillosis, Cirrhosis, Intensive care unit, Immunosuppression

## Abstract

**Background:**

Cirrhosis is not recognised as one of the main risk factors of invasive pulmonary aspergillosis (IPA), although its prevalence is increasing. The aim of our study was to identify factors for IPA in such patients with a positive *Aspergillus* sp. culture in respiratory samples and to evaluate its impact on outcome.

**Methods:**

We conducted a monocentric retrospective study between January 2005 and December 2015. All cirrhotic patients hospitalised in our liver ICU with a positive *Aspergillus* sp. respiratory sample were included. These patients were case-matched with cirrhotic patients without positive Aspergillus respiratory sample. Finally, the patients were classified as having putative aspergillosis or colonisation according to the criteria described previously.

**Results:**

In total, 986 cirrhotic patients were admitted to ICU during the study period. Among these, sixty patients had a positive *Aspergillus* sp. respiratory sample. Chronic obstructive pulmonary disease (COPD) comorbidity and organ supports were significantly associated with *Aspergillus* colonisation. Seventeen patients (28%) were diagnosed as proven or putative IPA and 43 were considered as colonised by *Aspergillus* sp. The median delay between ICU admission and an IPA diagnosis was 2 [2–24] days. Only COPD was predictive of the presence of IPA (OR 6.44; 95% CI 1.43–28.92; *p* = 0.0151) in patients with a positive *Aspergillus* sp. culture. The probability of in-hospital mortality was 71% in the IPA group versus 19% in the colonisation group (*p* = 0.0001).

**Conclusion:**

Patients with cirrhosis can be at risk of IPA, especially with COPD. Antifungal agents should be given as soon as possible mainly in cirrhotic patients with COPD.

## Introduction

Patients with cirrhosis are at risk of developing infection, sepsis and sepsis-induced organ failure [1]. Indeed, about 30% of cirrhotic patients admitted to an intensive care unit (ICU) have acquired or will acquire infections, most frequently involving the respiratory tract, urinary tract or ascites [2]. This high incidence of infection can partly be explained by cirrhosis-associated immune dysfunction. This state of immunodeficiency is a complex multifactorial process resulting from bacterial translocation, with the continuous stimulation of immune system cells, deficiencies in the complement system, the downregulation of monocytes and depressed neutrophil phagocytosis [3]. Recent studies have shown that some opportunistic infections, such as *Pneumocystis jirovecii* and cytomegalovirus infection, are seen in this specific population [4, 5]. Increasing numbers of fungal infections are also being observed [6, 7]. Among these pathogens, *Aspergillus* species, especially in respiratory samples, have emerged as an important cause of life-threatening infections in cirrhotic patients [8–13]. In immunocompromised patients, neutropenia and/or undergoing hematopoietic stem cell or solid organ transplantation, invasive pulmonary aspergillosis (IPA) is a major cause of morbidity and mortality [14]. Because of a delayed diagnosis due to a low index of suspicion, the same prognosis has been reported in cirrhotic patients than in this critically ill non-neutropenic population [9, 10, 15]. Its clinical and radiological signs are usually non-specific or absent [16]. Fungal cultures provide limited information because upper airway colonisation is common. Finally, galactomannan antigen test in serum and bronchoalveolar lavage (BAL) still needs to be validated in non-neutropenic populations [17].

Given the paucity of available of data, the aim of our study was therefore to identify factors for IPA in patients with a positive *Aspergillus* sp. culture in respiratory samples, to analyse different rules for the diagnosis and to evaluate its impact on outcome.

## Patients and methods

### Patients

This monocentric retrospective cohort study was conducted in the liver ICU (19 beds) at Henri Mondor University Hospital (a 900-bed, tertiary care institution for adults), between January 2005 and December 2015. In compliance with French law and regulations, the need for informed consent was waived. The Hospital’s Ethics Committee nevertheless approved the study, and the database was officially registered with the French Data Protection Authority (Commission Nationale Informatique et Liberté) (no. 1699340).

All patients included in this study were > 18 years of age, hospitalised in the liver ICU and had histologically proven or clinically diagnosed cirrhosis (e.g. portal hypertension with ascites, oesophageal varices or encephalopathy). For the purposes of this study, cirrhotic patients with inherited severe immunodeficiency, allogenic stem cells or who had undergone organ transplantation, were excluded.

### Diagnosis of invasive pulmonary aspergillosis (IPA)

Patients were eligible when they had evidence of *Aspergillus* sp. in at least one positive respiratory sample (sputum, tracheal aspirate, protected sample brush or BAL) specimen during the ICU course. The following factors contributed to the diagnosis of putative IPA or colonisation: (1) detection of *Aspergillus* by direct examination or culture, (2) analysis of a computed tomography (CT) scan with bilateral nodular infiltrates with central cavitation, halo sign or the air-crescent sign, (3) signs of lower respiratory tract infection or pleural rub, (4) *Aspergillus* galactomannan (GM) antigen determined in respiratory samples and/or serum using ELISA kit (Platelia Aspergillus, Bio-Rad Laboratories, Marne la Coquette, France) and (1,3)-β-d-glucan (BG) assay (Fungitell^®^, Cape Cod USA) only available in our institution since 2007.

All patients were reviewed blind by two intensivists, two mycologists and one radiologist and were classified according to Blot et al. for critically ill patients. The patients were classified as having proven or putative IPA or *Aspergillus* respiratory tract colonisation [18]. *Aspergillus* colonisation was defined as the presence of *Aspergillus* in bronchial secretions but without radiological, clinical or microbiological evidence of invasive infection.

A matched cohort analysis was performed to assess whether Aspergillosis colonisation impacts the survival of cirrhotic patients. Each patient with *Aspergillus* sp. with at least one positive respiratory sample (Aspergillus + group) was matched to two or if possible three comparative patients (patients hospitalised in liver ICU with at least one respiratory sample but without positive sample). These comparative studies were taken from a prospective cohort of patients admitted to the liver ICU in Henri Mondor Hospital during the period 2011–2017. Patients in both groups were matched according to gender, age (± 5 years) and mechanical ventilation.

### Data collection

The following parameters were collected for each patient: demographic characteristics [age, gender, reason for ICU admission, risk factors for invasive fungal infection (IFI), immune status and immunosuppressive drugs, malignancy]; scores for the severity of liver dysfunction, of which Child–Pugh score, MELD score, SOFA score, Grade of Acute-On-Chronic Liver failure as defined by the EASL-CLIF consortium/CANONIC study, i.e. patients with previously known chronic liver disease and at least one organ failure defined by the Clif-SOFA [19]; medical history (underlying diseases); clinical and laboratory findings and microbiological data; presentation and management of IPA; outcomes at hospital discharge and 1 year later were also collected.

### Statistical analysis

Continuous data were described as median [range]. The Chi-square test or Fisher’s exact test (categorical variables) and the Kruskal–Wallis or Mann–Whitney test (continuous variables) were used to compare demographic data based on the patient’s infection status, as appropriate. Logistic regression was used to identify factors associated with a diagnosis of IPA. No correction for multiple testing was performed, and therefore, univariate analyses should be interpreted with caution. All variables were considered for the multivariate logistic regression model if the *p* value was < 0.2 in the univariate analysis. When the composite score was tested, factors included in it were not further considered for multivariate analysis. Kaplan–Meier curves were generated, and a log-rank test was used to compare patients with IPA or colonisation. All statistical analyses were performed using SPSS (version 24; SPSS Inc, IBM, Chicago, IL, USA). A *p* value lower than 0.05 was considered to be statistically significant.

## Results

Between January 2005 and December 2015, 9176 patients were admitted to the liver ICU in our institution. Among these, 986 (10.7%) had histologically proven or clinically diagnosed cirrhosis. The median age in cirrhotic population was 57 ± 12.3 years; 71% were men and their liver disease was most frequently alcohol related. The cumulative incidences of ICU and hospital mortality were 29% (285 out of 986) and 37% (364 out of 986), respectively. Three hundred and forty-one patients (34.5%) required mechanical ventilation for at least 48 h.

### Risk factors for colonisation

A total of 362 samples have been performed in the two cohorts (positive *Aspergillus* and negative *Aspergillus*) with a rate of positivity of 17.4% (63/362 samples). Baseline characteristics did not differ between positive *Aspergillus* and negative *Aspergillus* except aetiology of cirrhosis (Table 1). Alcohol cirrhosis was significantly more frequent in negative *Aspergillus* patients than in others. Patients with chronic obstructive pulmonary disease (COPD) were more frequent in positive *Aspergillus* (38% vs. 14%, *p* = 0.001). The two groups did not differ regarding severity of illness (prognostic scores, laboratory data). Even if more positive *Aspergillus* patients fulfilled the criteria for Acute-On-Chronic Liver Failure, no significant difference was observed (72% vs. 59%, *p* = 0.09). Organ supports (catecholamine use, renal replacement therapy and liver support) were significantly associated with *Aspergillus* colonisation (Table 1).

**Table 1:** Demographic, clinical and laboratory characteristics of cirrhotic patients with (Aspergillus +) or without (Aspergillus −) positive culture of *Aspergillus* spp

Variables	Aspergillus + *n* = 60	Aspergillus – *n* = 148	*p* value
Age (years), mean (SD)	55.4 (12.6)	54.7 (11.5)	0.6
Gender (male), *n* (%)	51 (85)	125 (84)	0.38
Aetiology of cirrhosis, n (%)			< 0.001
Alcohol	25 (42)	106 (71)	
Viral	23 (38)	32 (22)	
Other	12 (20)	10 (7)	
Comorbidities, *n* (%)			
COPD	23 (38)	20 (14)	0.001
Immunosuppressive treatment	15 (25)	22 (15)	0.08
Ascites	24 (40)	47 (32)	0.82
Life support *n* (%)			
Mechanical ventilation	31 (52)	77 (52)	> 0.99
Catecholamine	30 (50)	50 (34)	0.03
Renal replacement therapy	13 (22)	13 (9)	0.01
Liver support	7 (12)	5 (3.4)	0.02
Laboratory data, mean (SD)			
INR	2.01 (1.64)	2.4 (2)	0.23
Serum bilirubin (µmol/l)	131 (159)	151(165)	0.056
Serum creatinine (µmol/l)	155 (151)	149 (131)	0.98
Serum sodium (mmol/l)	135 (5.3)	134 (7.7)	0.89
Prognostic score, mean (SD)			
Child–Pugh score	11.2 (2.8)	10.6 (2.3)	0.15
MELD score	21 (11.2)	22,6 (9.8)	0.25
SAPS II score	46 (20)	44 (19,7)	0.68
SOFA score	9.8 (7.3)	8.6 (4.9)	0.55
ACLF grade *n* (%)			0.26
ACLF grade 0	17 (28)	60 (41)	
ACLF grade 1	11 (18)	30 (20)	
ACLF grade 2	9 (15)	19 (13)	
ACLF grade 3	23 (38)	39 (26)	
Outcome, *n* (%)			
Hospital mortality	20 (33)	52 (35)	0.8
Liver transplantation	5 (8)	13 (9)	0.91

Significant parameters (p value < 0.05)

*IPA* invasive pulmonary aspergillosis, *COPD* Chronic Obstructive Pulmonary Disease, *MELD* Model of End-Stage Liver Disease, *ACLF* Acute-On-Chronic Liver Failure, *ICU* Intensive Care Unit, *SAPS II* Simplified Acute Physiology Score

### Positive *Aspergillus* patients characteristics

**Fig. 1:**
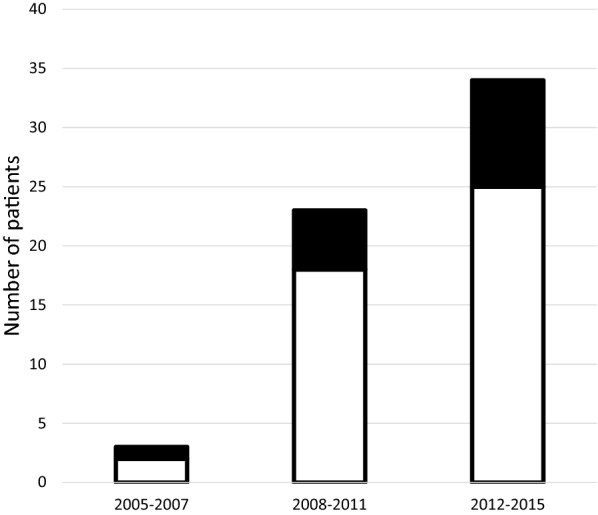
Individual cases of invasive pulmonary aspergillosis (solid bars) and *Aspergillus* spp. colonisation (open bars) reported from 2005 to 2015 (*n* = 60)

Finally, 60 patients fulfilled the study criteria (positive culture of *Aspergillus* sp.) and were included in this monocentric retrospective analyse. With the increase in the number of patients hospitalised in liver ICU between 2005 and 2015, the occurrence of IPA and/or respiratory tract colonisation by *Aspergillus* in cirrhotic patients has increased in the past 10 years (Fig. 1). The demographic features, risk factors and outcome data of the 60 patients included are summarised in Table 2. Overall, 85% of the patients were male and their median age was 55.4 ± 12.6 years. Their cirrhosis was alcohol related (42%) or viral related (38%). The most common primary diagnoses at ICU admission were sepsis (*n* = 24, 40%), acute variceal bleeding (*n* = 17, 28%) or hepatic encephalopathy (*n* = 12, 20%). Twenty patients (33%) were admitted with respiratory failure. Among the patients with alcoholic cirrhosis, 11 (18%) had histologically proven severe acute alcoholic hepatitis and had been treated with corticosteroids (with a duration of 7–13 days), during the 3 months prior to their hospitalisation in the liver ICU. In addition, four patients received immunosuppression treatment (steroids for autoimmune cirrhosis *n* = 2 and azathioprine for psoriasis *n* = 1 or CREST syndrome *n* = 1). The Child–Turcotte–Pugh scores of all patients were B or C (22% and 78%, respectively). The mean MELD score and SOFA score were 21 ± 11.2 and 9.8 ± 7.3, respectively. Forty-three patients (72%) fulfilled the criteria for Acute-On-Chronic Liver Failure. No patient had a history of fungal infection or fungal prophylaxis at the time of diagnosis.

**Table 2:** Demographic, clinical and laboratory characteristics of patients with positive culture of *Aspergillus* spp., invasive pulmonary aspergillosis and *Aspergillus* spp. colonisation

Variables	Whole population *n* = 60	IPA *n* = 17	*Aspergillus* spp. colonisation *n* = 43	*p* value
Age (years), mean (SD)	55.4 (12.6)	56 (14.5)	55.2 (11.8)	0.64
Gender (male), *n* (%)	51 (85)	15 (88)	36 (84)	0.65
Aetiology of cirrhosis, *n* (%)				0.60
Alcohol	25 (42)	8 (47)	17 (40)	
Viral	23 (38)	7 (41)	16 (37)	
Other	12 (20)	2 (12)	10 (23)	
Severe alcoholic hepatitis, *n* (%)	11 (18)	6 (35)	5 (12)	0.06
Comorbidities, *n* (%)				
Diabetes	14 (23)	2 (12)	12 (28)	0.18
COPD	23 (38)	11 (65)	12 (28)	0.008
Smoking history	23 (38)	6 (35)	17 (40)	0.76
Heart disease	3 (5)	0 (0)	3 (7)	0.26
Immunosuppressive treatment	15 (25)	7 (41)	8 (19)	0.09
Ascites	24 (40)	9 (53)	15 (35)	0.19
Encephalopathy	17 (28)	9 (53)	8 (19)	0.007
Life support *n* (%)				
Mechanical ventilation	31 (52)	12 (71)	19 (44)	0.06
Catecholamine	30 (50)	12 (71)	18 (42)	0.04
Renal replacement therapy	13 (22)	7 (41)	6 (14)	0.02
Liver support	7 (12)	4 (24)	3 (7)	0.07
Laboratory data, mean (SD)				
INR	2.01 (1.64)	2.2 (1.5)	1.9 (1.7)	0.56
Serum bilirubin (µmol/l)	131 (159)	172 (169)	115 (154)	0.16
Serum creatinine (µmol/l)	155 (151)	180 (151)	145 (152)	0.14
Serum sodium (mmol/l)	135 (5.3)	134 (5.8)	135 (5.3)	0.61
Albumin (g/L)	31 (8)	34 .4 (8.6)	30.9 (7.9)	0.20
Leucocytes (/mm3)	10.9 (8.4)	13.8 (12.4)	9.9 (6.3)	0.69
Platelets (/mm3)	123 (81)	109 (69)	127 (85)	0.66
Prognostic score, mean (SD)				
Child–Pugh score	11.2 (2.8)	11.8 (3.1)	10.8 (2.6)	0.17
SAPS II	46 (20)	48 (25)	45 (18)	0.32
MELD score	21 (11.2)	23.2 (13.3)	20.1 (10.4)	0.44
SOFA score	9.8 (7.3)	13.4 (8.5)	8.3 (6.4)	0.002
ACLF grade *n* (%)				0.17
ACLF grade 0	17 (28)	4 (24)	13 (30)	
ACLF grade 1	11 (18)	1 (6)	10 (23)	
ACLF grade 2	9 (15)	2 (12)	7 (16)	
ACLF grade 3	23 (38)	10 (59)	13 (30)	
Outcome, *n* (%)				
Hospital mortality	20 (33)	12 (71)	8 (19)	< 0.001
One-year mortality	41 (68)	14 (82)	27 (63)	0.14
Liver transplantation	5 (8)	1 (6)	4 (9)	0.66

Significant parameters (p value < 0.05)

*IPA* invasive pulmonary aspergillosis, *COPD* chronic obstructive pulmonary disease, *MELD* Model of End-Stage Liver Disease, *ACLF* Acute-On-Chronic Liver Failure, *ICU* intensive care unit, *SAPS II* Simplified Acute Physiology Score

### Invasive pulmonary aspergillosis (IPA) versus respiratory tract *Aspergillus* colonisation

*Aspergillus* sp. were isolated from 60 cirrhotic patients from BAL specimens (*n* = 52, 87%) or bronchial aspirates (*n* = 8, 13%). The most common *Aspergillus* species were *A. fumigatus* (*n* = 45, 75%), *A. flavus* (*n* = 4, 6%) and *A. nidulans* (*n* = 5, 8%). In five cases, the respiratory cultures yielded two types of *Aspergillus* sp. (*A. fumigatus *+ *A. flavus* (*n* = 2), *A. fumigatus* + *A. nidulans* (*n* = 1), *A. versicolor* + *A. nidulans* (*n* = 1) and *A. versicolor* + *A. niger* (*n* = 1)). The clinical and biological characteristics of patients with IPA are summarised in Table 3.

**Table 3:** Clinical, laboratory parameters finding and antifungal therapy of patients with liver cirrhosis and invasive pulmonary aspergillosis

Variables	IPA (*n* = 17)
Proven IPA	2
Probable IPA	15
Clinical characteristics	
Fever	11
Cough	15
Hemoptysis	1
Laboratory parameters	
Leucocyte counts × 109/L, mean (SD)	13.8 (12.4)
Mycological culture	
*A. fumigatus*	16
*A. flavus*	1
Antifungal therapy	
Voriconazole	6
Caspofungin	5
Liposomal amphotericin B	2

*IPA* invasive pulmonary aspergillosis

Seventeen IPA were classified as proven (*n* = 2) or putative (*n* = 15). The sites of infection included the lungs only (*n* = 15) or the lungs and central nervous system (*n* = 2). These last two patients had multiple cerebral abscesses on cerebral CT and pulmonary nodules. The final diagnosis was performed by pulmonary biopsies, corresponding to the two IPA classified as proven. Eleven patients with IPA (65%) had fever, 15 had a cough, and one patient suffered from haemoptysis. In the two proven IPA patients, *A. fumigatus* was diagnosed from a lung biopsy. The putative IPA was diagnosed by a combination of a positive culture, compatible signs, radiological imaging and mycology criteria (galactomannan (GM) positivity (serum and/or BAL) or serum 1,3-β-d-glucan (Tables 4, 5).

**Table 4:** Chest CT scan in patients categorised as having proven and putative aspergillosis or *Aspergillus* colonisation

	All (*n* = 60)	Proven/putative aspergillosis (*n* = 17)	*Aspergillus* colonisation (*n* = 43)	*p* value
Pulmonary infiltrates	24	11	13	0.02
Alveolar consolidation	15	7	8	0.09
Lung nodules	13	7	6	0.03
Ground-glass opacities	3	2	1	0.2
Cavitation	1	0	1	> 0.99
Halo sign	0	0	0	/
Pleural effusion	17	10	17	0.2

**Table 5:** Serum and bronchoalveolar lavage fluid galactomannan and 1,3-β-d-glucan in patients categorised as having proven and putative aspergillosis or *Aspergillus* colonisation

	All	Proven/putative aspergillosis (*n* = 17)	*Aspergillus* colonisation (*n* = 43)	*p* value
Positive serum galactomannan	14/42	10/12 (83%)	4/30 (13%)	< 0.001
Positive BAL galactomannan	3/11	3/5 (60%)	0/6 (0%)	0.06
Serum 1,3-β-d-glucan > 80 pg/ml	12/23	6/8 (75%)	6/13 (46%)	0.36

Numbers of patients with a positive measurement/numbers of patients with measurement performed

Chest CT scan was obtained in 95% (*n* = 57/60) of patients during the ICU stay. Pulmonary infiltrates and nodules were more frequently in patients categorised as having proven/putative aspergillosis than in those with *Aspergillus* sp. colonisation (Table 4). Among the 60 patients with positive *Aspergillus* sp. in respiratory samples, 42 (70%) and 11 (18%) had a GM measurement taken in serum and BAL fluid, respectively. Serum GM measurements were significantly different between patients with proven/putative aspergillosis and those with *Aspergillus* sp. colonisation (*p* < 0.001). Among patients with positive serum GM, three patients were or had been treated with tazocilline (vs. two in the *Aspergillus* sp. colonisation group and one in the proven/putative aspergillosis group). In contrast, when GM is performed in BAL fluid, no difference was observed, probably related to the limited number of GM measurements taken in BAL (11 out of 60 patients). Also, serum BDG measurements, from 2009 to 2015, were not significantly different between both groups (*p* = 0.36) (Table 5).

Cases of IPA were diagnosed after a median delay of 12 [2–24] days following admission to the ICU. Four patients died before the culture results were available and had not been treated with any antifungal treatment. The other patients with IPA were treated with voriconazole (*n* = 6), caspofungin (*n* = 5) or liposomal amphotericin B (*n* = 2).

Risk factors for IPA, such as chronic obstructive pulmonary disease (COPD) (*p* = 0.008) or encephalopathy (*p* = 0.007), meant that the patients had more frequently received organ support (renal replacement therapy or liver support) and had a higher SOFA score at ICU admission (*p* = 0.002). Under multivariate regression logistic analysis, only COPD was predictive of the presence of IPA (OR 6.44; 95% CI 1.43–28.92; *p* = 0.0151) in patients with a positive *Aspergillus* sp. culture.

### Outcomes

**Fig. 2:**
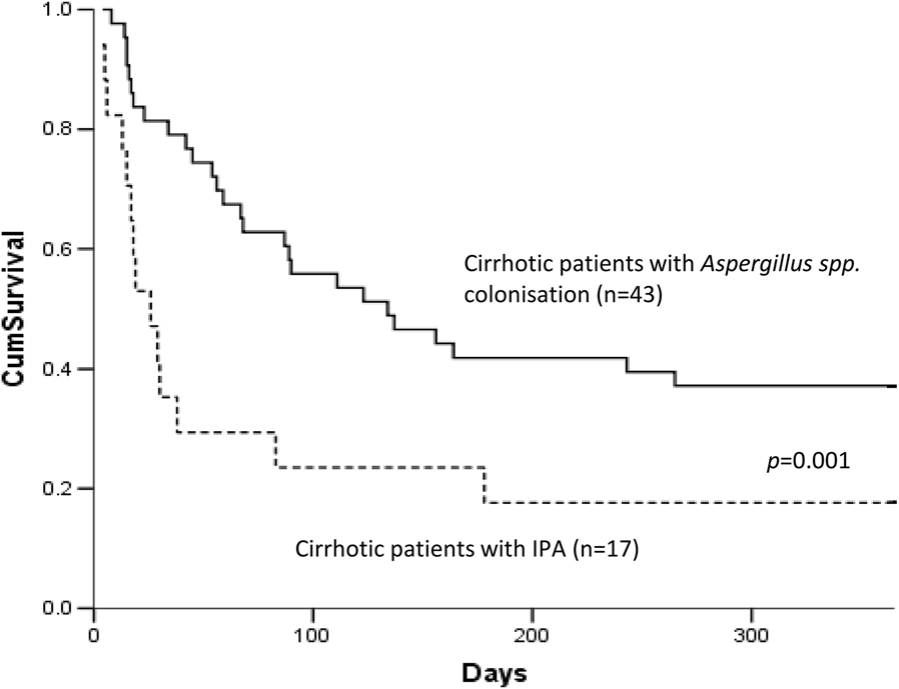
Kaplan–Meier survival curve of 17 cirrhotic patients with IPA and 43 patients with *Aspergillus* spp. colonisation. Curves were compared using log-rank test

There was no difference between positive *Aspergillus* and negative *Aspergillus* in respiratory samples of patients regarding in-hospital mortality and number of liver transplantation (Table 1). Among the patients with positive *Aspergillus*, the overall hospital and 1-year mortality rates were 33% (20 out of 60 patients) and 68% (41 out of 60 patients), respectively (Fig. 2). The probability of hospital mortality was significantly higher among patients with IPA than in patients with colonisation [12/17 (71%) vs. 8/43 (19%), *p* = 0.0001]. However, no statistically significant difference in the 1-year mortality rate was observed between these two groups [14/17 (82%) vs. 27/43 (63%); *p* = 0.14]. In patients with IPA, 12 patients died during the hospital stay, among whom four before the diagnosis. For others, the time span between the diagnosis and death was 12 days (2–28). The main cause of death was multiple organ failure secondary to septic shock. Among these patients, five patients were listed for liver transplantation, but only one was transplanted (28 days after the IPA diagnosis) and then treated with voriconazole for 1 year.

## Discussion

This study documents the epidemiology of aspergillosis in cirrhotic patients over an 11-year period. We observed that (1) *Aspergillus* sp. lower respiratory tract specimen culture was positive in 6% of cirrhotic patients admitted to the liver ICU, and (2) COPD was a risk factor for IPA in patients when respiratory samples were positive in culture for *Aspergillus* sp. In addition, our results led us to consider this infection as more common and as being associated with high hospital and 1-year mortality rates.

IPA is an infection that mainly occurs in patients with neutropenia, receiving immunosuppressive therapy in the context of solid organ transplant or haematological malignancies. Aside from these high-risk groups, many cases of IPA have been reported in non-traditional hosts, especially cirrhotic patients [8, 9, 11]. In this era of advanced organ-targeted therapy in critical care, we know that the key to improve ICU survival in cirrhotic patients is to adopt a more aggressive management [20, 21]. For some years now, more patients with cirrhosis are being admitted to ICU, pathophysiology of cirrhosis is better understood, and new therapies are available [22]. In our experience, the number of cirrhotic patients admitted in ICU increases, in an order of 5–10% each year. Our findings concerning the incidence of aspergillosis colonisation are consistent with previously published data [12, 13, 23, 24]. Indeed, the rate of fungal colonisation can reach 25% in critically ill cirrhotic patients, with *Aspergillosis* up to 3% [25]. Further, the data regarding the incidence of IPA ranged from 0.288% in a tertiary centre in China [8] to 16% among patients with severe alcoholic hepatitis [10]. According to our result, the alcoholic cirrhotic patients with organ failure and having a COPD comorbidity is at high risk of *Aspergillus* colonisation.

In a context of *Aspergillus* sp*.-*positive cultures, discriminating between colonisation and infection remains challenging. According to the European Organisation for Research and Treatment of Cancer/Mycosis Study Group (EORTC/MSG), IPA is categorised as a proven, probable or possible fungal infection [26]. This categorisation requires histopathological evidence or the presence of a combination of host factors, clinical and radiological features and positive mycology. However, in most ICU patients, the entry criterion is an positive *Aspergillus* culture in lower tract. A simple clinical algorithm has thus been proposed to discriminate *Aspergillus* sp. respiratory tract colonisation from putative IPA [18]. During our study, we chose to use this algorithm to evaluate the incidence of IPA in cirrhotic patients and found that among 60 patients with an *Aspergillus*-positive culture, nearly one in three had an IPA. Because of the high mortality associated with IPA, it could be important to identify the risk factors for this condition. Early diagnosis and prompt antifungal therapy may improve the outcome. Also, a history of COPD, in our study, was the only factor associated with IPA in cirrhotic patients with positive *Aspergillus* cultures in respiratory samples. A baseline MELD score > 24 and corticosteroid therapy have both been identified as risk factors for IPA [10]. In our experience, neither the mean MELD score nor the number of patients with a MELD score > 24 were different between the group with IPA and those with the colonised group. In addition, the notion of immunosuppressive treatment (*p* = 0.09) was not IPA risk factors. These conflicting results may be due to the population studied. Gustot et al. [25] included all cirrhotic patients and compared patients with and without IPA, while we studied cirrhotic ICU patients with a positive *Aspergillus* culture and compared those with IPA and those only colonised. However, even though it was not statistically significant, more patients in the IPA group received immunosuppressive treatment (41% vs. 19%) and the need of mechanical ventilation was higher (71% vs. 44%).

We found that patients with COPD have an increasingly recognised risk of developing IPA [27–29]. This can be explained by the fact that cirrhotic and COPD patients have a greater susceptibility to IPA, as impairment of the defence mechanisms in the airways, repeated short-term use of corticosteroids, frequent hospitalisation and broad-spectrum antibiotics with pathogen selection and conditions for comorbidity. We did not find an association between IPA and the presence of other pathogens (such as H1N1 infection). This result may be at odds with our previous observation [29]. Indeed, in seven patients with liver disease an association between co-infection by *A. fumigatus* and *S. maltophilia* has been observed [30]. However, in this previous work, we were not able to distinguish between *S. maltophilia* infection and colonisation, which made it difficult to reach conclusions on the specific role of the bacterium in the respiratory pathology.

The mortality rate in cirrhotic patients with IPA exceeded 50% in an overview report [22, 31] even though there has been a trend in recent years towards a decline [9]. In our series, hospital mortality was higher among IPA patients than in those colonised by *Aspergillus*. However, this difference was not observed at 1 year, probably because the mid-term prognosis of cirrhotic patients with organ failure and hospitalised in the ICU is poor at 1 year, with or without infection [19]. The poor prognosis of IA in cirrhotic patients may be explained by a higher immunosuppression state in patients with end-stage liver disease, the late diagnosis or/and lower efficiency of antifungal therapy used in the context of multiorgan failure.

More studies are necessary in order to improve estimates concerning the survival rate of patients with IPA and cirrhosis. Meanwhile, because IPA in cirrhotic patients is associated with high mortality, a prophylactic strategy, especially in patients with severe alcoholic hepatitis that have an incidence of 15.8% [10], could be proposed as soon as when *Aspergillus*-positive samples are found, and might be more effective than a therapeutic approach. This also needs to be evaluated.

The retrospective nature of our study was its first limitation, with a certain bias affecting the reports (e.g. a greater tendency to search for *Aspergillus* over time). Indeed, we observed a rise in the incidence of *Aspergillus* respiratory tract colonisation and IPA as the study period advanced. Our study is not supposed to describe the epidemiology of aspergillosis in cirrhotic but describe the characteristics and outcome of cirrhotic patients with positive *Aspergillus* culture. If we consider that patients without respiratory samples do not have IPA, a positive culture of *Aspergillus* spp. and proven or putative IPA were found in, respectively, 6% and 1.7% of cirrhotic patients admitted to the ICU. Secondly, although cirrhosis is associated with a state of immunodeficiency, it is not recognised as a risk factor according to the EORTC/MSG criteria. In ICU patients, Blot et al. observed that only 41% were immunocompromised and proposed to use as criteria either the presence of a host risk factor or a semi-quantitative *Aspergillus*-positive culture of BAL fluid. In our study, 15 patients had host risk factors (immunosuppression treatment in the severe hepatitis alcoholic context or autoimmune cirrhosis [10]) and five had an *Aspergillus* sp*.-*positive culture of BAL fluid in the IPA group. However, in light of a review of aspergillosis cases described in cirrhotic patients [31], we believe that cirrhosis could be seen as a host risk factor. In our study, however, even if cirrhosis had been considered as a host risk factor in the colonisation group, no patients had compatible signs or symptoms or/and abnormal medical imaging findings that enabled a diagnosis of IPA. Thus, we think that decompensated cirrhosis should be added to the list of host risk factors in the algorithm developed by Blot et al. [18].

In conclusion, our study was able to confirm that the occurrence of IPA or respiratory tract colonisation by *Aspergillus* sp. in cirrhotic patients is increasing in the past 10 years, probably in relation to the number of cirrhotic patients admitted in liver ICU. In addition, following the publication of Gustot et al. [10], we were sensitised to this infection, with more frequent research. Moreover, this *Aspergillus* infection is associated with higher mortality. Based on our results, we recommend that cirrhotic patients with sepsis admitted to ICUs should be screened for fungal infections (bronchial aspiration, chest CT, biological markers, etc.), especially in cases of strong suspicion (hepatitis alcoholic, fewer persistence, etc.). This could also be useful to obtain an early diagnosis of IPA and a swift treatment of this complication, particularly in cirrhotic patients with COPD.

## Abbreviations

IPA: invasive pulmonary aspergillosis
ICU: Intensive care unit
MELD: Model for End-Stage Liver Disease
COPD: chronic obstructive pulmonary disease
BAL: bronchoalveolar lavage
CT: computed tomography
BG: 1,3-β-d-glucan
IFI: invasive fungal infection
ACLF: Acute-on-Chronic Liver Disease
GM: galactomannan
